# Computational Assessment of Chito-Oligosaccharides Interactions with Plasma Proteins

**DOI:** 10.3390/md19030120

**Published:** 2021-02-24

**Authors:** Diana Larisa Roman, Vasile Ostafe, Adriana Isvoran

**Affiliations:** Department of Biology-Chemistry and Advanced Environmental Research Laboratories, West University of Timisoara, 300223 Timisoara, Romania; diana.roman@e-uvt.ro (D.L.R.); vasile.ostafe@e-uvt.ro (V.O.)

**Keywords:** chito-oligosaccharides, human serum albumin, α-1-glycoprotein

## Abstract

It is widely rec ognized that chitin and chitosan are potential sources of bioactive materials and that their oligosaccharides reveal various biological activities (including antimicrobial) that are correlated with their structures and physicochemical properties. This study uses the molecular docking approach to assess the interactions of small chito-oligosaccharides (MW< 1500 Da) with plasma proteins in order to obtain information regarding their fate of distribution in the human organism. There are favorable interactions of small chito-oligomers with plasma proteins, the interactions with human serum albumin being stronger than those with α-1-acid glycoprotein. The interaction energies increase with increasing the molecular weight, decrease with increasing deacetylation degrees and are reliant on the deacetylation pattern. This study could inform the application of chito-oligosaccharides with varying molecular weights, degrees, and patterns of deacetylation in human health.

## 1. Introduction

Chitin is an important natural polymer that is largely exploited from marine sources (usually crustaceans, shrimp, and crabs) [1]. Depending on the source of obtaining, there are three polymorphic crystalline structures of chitin that can be produced: α-chitin, β-chitin, and γ-chitin. When crabs and shrimps are the sources, usually the isomorph α-chitin is obtained, while the isomorph β-chitin is acquired from the squid bones and γ-chitin is usually obtained from insects. The three isomorphs reveal distinct characteristics. The α-chitin has a compact crystalline structure with antiparallel chains of N-acetyl glucosamine supporting strong intersheet and intrasheet hydrogen bonding. The β-chitin also has a crystalline structure but with parallel chains of N-acetyl glucosamine supporting weak hydrogen bonding. In the case of γ-chitin, two chains run in one direction and the another chain runs antiparallel to them [2]. These allomorphic variants of chitin present distinct properties, β-chitin revealing higher solubility, reactivity, and swelling capacity. These findings suggest that the source of chitin may influence its applications in biomedical and pharmacological industries [3]. The most important derivative of chitin is chitosan that is obtained by chemical hydrolysis or enzymatic deacetylation of chitin. Consequently, the dissimilarity between chitin and chitosan polymers is expressed in the acetyl content: chitin exclusively contains N-acetyl-D-glucosamine (GlcNAc or A) units and chitosan encompasses both D-glucosamine (GlcN or D) and N-acetyl-D-glucosamine units [4]. The content of deacetylated units in the polymer defines the deacetylation degree (DD). There is not a strict delimitation between the chitin and chitosan nomenclature, usually a polymer with DD < 50% is called chitin and if DD > 50%, the polymer is called chitosan [1]. The properties of chitin and chitosan are quite different. Chitin is highly hydrophobic being insoluble in water and in many organic solvents, whereas chitosan is soluble in diluted acids. Additionally, the nitrogen content of chitin depends on the deacetylation degree and fluctuates from 5 to 8%, while in chitosan the nitrogen is mostly in the form of primary aliphatic amino groups conducting to specific reactions for amines, N-acylation and Schiff reaction. These reactions allow to easily obtain chitosan derivatives [5]. Chitin, and especially its derivative chitosan, have numerous applications in many fields: biomedical materials and pharmacological industry, food industry, cosmetics, and waste management [6,7,8]. The biomedical applications are based on the specific properties of chitin and chitosan: anti-bacterial, anti-microbial, anti-fungal, anti-oxidant, biocompatibility, biodegradability, and non-toxicity for both humans and environment [4,9,10].

The limitations concerning the biomedical applications of chitin and chitosan are due to their higher viscosity and low solubility in neutral and basic environments [11]. In order to reduce these limitation, derivatives of chitosan [7,12,13] and/or chito-oligosaccharides (COs) characterized by increased solubility and lower viscosity have been obtained [14]. COs containing both GlcN and GlcNAc and having maximum 10 monomers are considered as water soluble [15]. These oligomers reflect various medical applications: anti-microbial, anti-inflammatory, anti-oxidant, anti-tumoral, immunostimulatory, anti-hypertension, anti-obesity, and anti-Alzheimer [15,16,17]. Moreover, the use of chitosan as a drug delivery system and/or in tissue engineering may conduct to low-molecular degradation products, the chito-oligosaccharides, released under the influence of enzymes found in the fluids of the human organism. Consequently, small COs arrive in the human organism by volunteer intake or as degradation products of chitosan used for various medical purposes.

Different COs are characterized by the following parameters: molecular weight (MW), deacetylation degree (DD), and distribution of glucosamine residues in the chain (deacetylation pattern, DAP). Pharmaceutical actions of COs proved to be strongly dependent on their physicochemical properties [18]. A computational study performed by our group [19] showed that COs reveal promising pharmacological profiles and limited toxicological effects on humans, regardless of MW, DD, and DAP. According to this study, the possible toxicological effects of COs in the human organism consist in the inhibition of the organic anion transporting peptides OATP1B1 and/or OATP1B3, a small probability of affecting the androgen receptor, a low potential of cardiotoxicity, and the possibility of COs characterized by high DD to produce phospholipidosis [19]. However, it is necessary that these predictions of the computational studies are further verified by experiments.

Among absorption, distribution, metabolism, excretion, and toxicity (ADMET) properties of molecules, the plasma-protein binding is one of the most important as it affects transport and release of molecules. To the best of our knowledge, specific literature does not contain information about the distribution of COs in human organism. Distribution of chemicals depends on their ability to bind to plasma proteins and computational studies may have a valuable contribution to evaluate the interactions of COs with these proteins. Human serum albumin (HSA) and α-1-acid glycoprotein (AGP) are the major plasma proteins that are able to bind drugs and other bioactive compounds and to regulate the disposition and affect the fate of distribution of chemical compounds in the human organisms. These two proteins have strong effect on pharmacokinetics and pharmacodynamics of the chemical compounds. Consequently, the aim of this study is to assess the interactions of small COs with the two major plasma proteins using the molecular docking approach. We predict the binding poses and interacting energies and we also assess the influence of the MW, DD, and DAP on these interactions with consequences on regulation of COs disposition in the human organism. 

## 2. Results

### 2.1. Properties of Small Chito-Oligosaccharides Considered in This Study

The chito-oligosaccharides considered in this study are presented in Table 1 together with the values of their molecular weights.

### 2.2. Analysis of the Structural Files of Plasma Proteins

**Figure 1:**
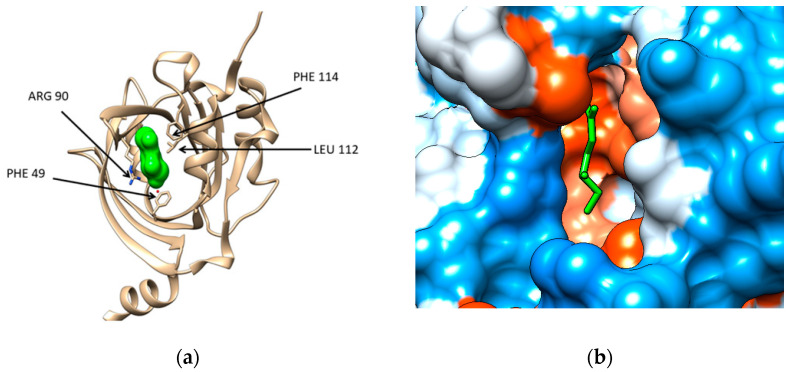
(**a**) Structure of the human α-1-acid glycoprotein (brown ribbon) in complex with the ligand (2R)-2,3-dihydroxypropyl acetate (green solid surface), Protein data Bank (PDB) code entry 3KQ0. The residues interacting with the ligand are emphasized: PHE 49, ILE 88 (not seen being behind the ligand), ARG 90, LEU112, and PHE114; (**b**) Illustration of the hydrophobicity surface of the binding cavity of α-1-acid glycoprotein: blue regions are hydrophilic and orange regions are hydrophobic (dodger blue for the most hydrophilic residue to white at 0.0 and orange red for the most hydrophobic residue) and the ligand is revealed in green sticks.

The structural files of the two major plasma proteins have been extracted from the Protein Data Bank (for details see Section 4). For the α-1-acid glycoprotein, we have considered the structural file with the PDB ID 3KQ0. It corresponds to a crystallographic structure of the AGP protein in complex with (2R)-2,3-dihydroxypropyl acetate [20]. The residues of AGP interacting with the ligand are PHE 9, ILE 8, ARG 90, LEU 112, and PHE 114 (Figure 1a). Chimera computational tool has been used to compute the hydrophobicity surface of AGP and Figure 1b reveals that the binding cavity of this protein is hydrophobic with a polar patch near to the entrance (Figure 1b).

**Figure 2:**
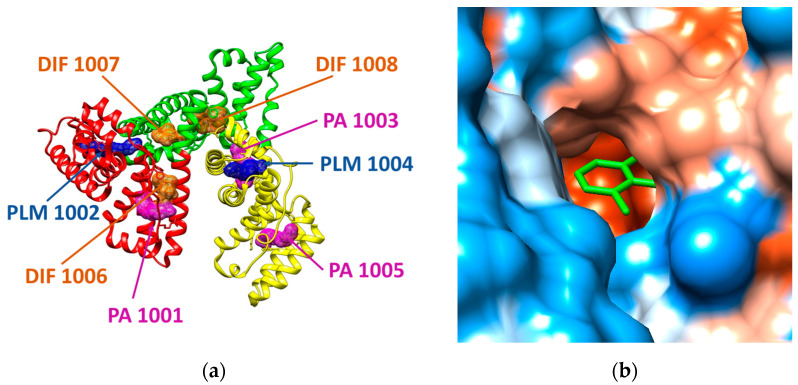
(**a**) Structure of human serum albumin (ribbon colored by domains: domain I is presented in red, domain II is reveled in green, domain III is presented in yellow) in complex with three molecules of palmitic acid (PLM, mesh surface magenta), three molecules of diclofenac (DIF, mesh surface brown), and two molecules of pentadecanoic acid (PA, mesh surface blue). (**b**) Illustration of the hydrophobicity surface of the binding cavity of DIF 1007 molecule: blue regions are hydrophilic and orange regions are hydrophobic (dodger blue for the most hydrophilic residue to white at 0.0 and orange red for the most hydrophobic residue) and the ligand is revealed in green sticks.

In the case of human serum albumin (HSA), the structural file with the PDB ID 4Z69 has been considered. This structural file corresponds to a dimer, but in our molecular docking study we only used the A chain, its structure being illustrated in Figure 2. The A chain of the structural file corresponds to the HSA in complex with several ligands: three molecules of diclofenac (DIF 1006, DIF 1007, and DIF 1008), three molecules of pentadecanoic acid (PA 1001, PA 1003, PA 1005), and two molecules of palmitic acid (PLM 1002, PLM 1004) [21]. HSA has three α-helical structural domains: domain I (residues 1–95, colored in red in Figure 2a), domain II (residues 196–83, colored in green in Figure 2a), and domain III (residues 384–585, colored in yellow in Figure 2a), each domain being divided into two subdomains (A and B) [22]. Domains II and III both have hydrophobic pockets commonly containing hydrophobic and positively charged residues and being able to accommodate a wide range of chemical compounds [22].

The structural file 4Z69 illustrates that there are two different binding sites for diclofenac molecules (DIF), one DIF molecule is positioned at the domain IB (DIF 1006) and the other two DIF molecules are situated in the hydrophobic cavity of the domain IIA, one in the main compartment (DIF 1007) and the other in the side compartment of the hydrophobic cavity (DIF 1008). One pentadecanoic acid (PA 1001) molecule co-binds with DIF in the subdomain IB and the other two molecules bind to the subdomains IIIA (PA 1003) and IIIB (PA 1005), respectively. One palmitic acid (PLM) molecule binds to IA subdomain (PLM 1002) and the other PLM molecule binds to IIIA subdomain (PLM 1004). 

The outcomes of the molecular docking study reveal that the most favorable binding modes for the investigated COs correspond to the region of the protein where one of the diclofenac molecules (DIF 1007) is bound in the crystallographic structure (see further). The binding cavity of DIF 1007 molecule reveals a high hydrophobicity, but there are polar residues in the inner surrounding and at the entrance of the cavity as it is illustrated in Figure 2b. The amino acids interacting with DIF 1007 molecule are LYS 199, TRP 214, ARG 218, LEU 219, ARG 222, ILE 264, and SER 287 (data not shown).

### 2.3. Molecular Docking Study

**Figure 3:**
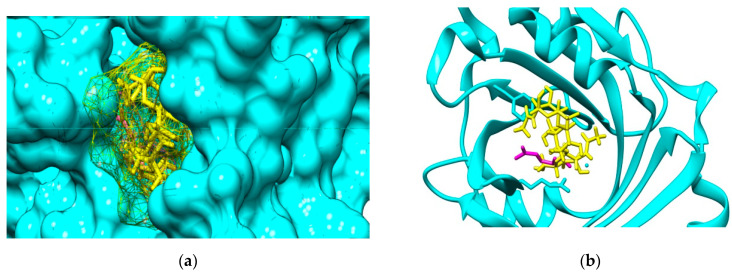
(**a**) Binding cavity of α-1-acid glycoprotein AGP (cyan solid surface) accommodating the ligand (2R)-2,3-dihydroxypropyl acetate (magenta sticks) and GlcNAc-GlcN-GlcNAc molecule (ADA, yellow mesh surface). (**b**) Detail of the positioning of the most favorable binding mode of GlcNAc-GlcN-GlcNAc (ADA yellow sticks) to AGP (cyan backbone) by comparison to the positioning of the ligand (2R)-2,3-dihydroxypropyl acetate (magenta sticks).

The molecular docking outcomes illustrate that investigated COs are able to bind to both AGP and HSA plasma proteins. For the interactions of COs with AGP, the most favorable binding mode corresponds to the position of the (2R)-2,3-dihydroxypropyl acetate, the ligand that is present in the crystallographic structure. Figure 3 illustrate the result of the molecular docking study for the binding pose corresponding to the highest interaction energy of GlcNAc-GlcN-GlcNAc (ADA) oligomer with AGP. This binding pose matches to the cavity of AGP accommodating the ligand (2R)-2,3-dihydroxypropyl acetate. 

**Figure 4:**
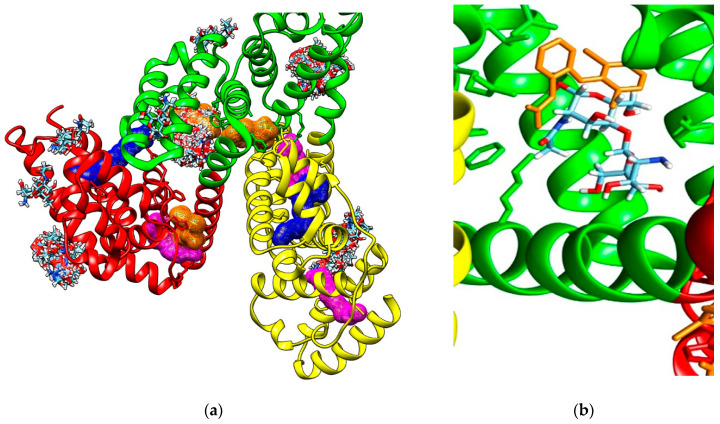
(**a**) Binding modes of GlcNAc-GlcN oligomer (AD, sticks colored upon atom type: carbon in light blue, nitrogen in dark blue, oxygen in red and hydrogen in white) to human serum albumin (HSA). Most binding modes correspond to the diclofenac 1007 molecule (brown mesh surface) binding site in the subdomain IIA (green cartoon); (**b**) Positioning of the GlcNAc-GlcN (DA) oligomer in the cavity of the subdomain IIA of HSA (green ribbon) by comparison to the DIF 1007 molecule (brown sticks).

Molecular docking study also reveals that investigated COs are able to bind to the hydrophobic cavity of the domain IIA of HSA, similar with DIF 1007 molecule. Figure 4 illustrate all the binding modes of GlcNAc-GlcN (AD) oligomer to HSA (Figure 4a) and the binding mode corresponding to the highest interaction energy respectively (Figure 4b). 

**Figure 5:**
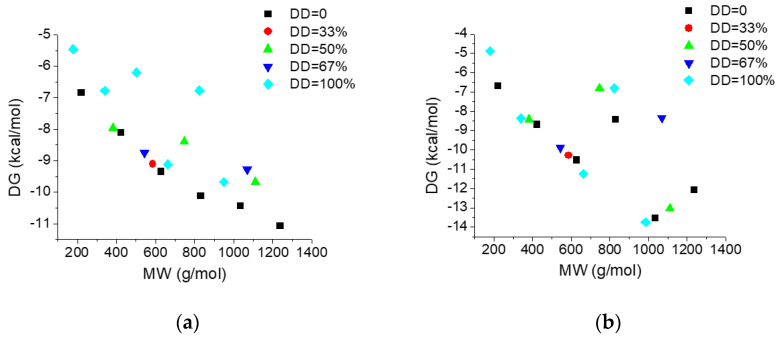
Dependence of the interacting energies of chito-oligosaccharides (COs) with AGP (**a**) and HSA (**b**) on the molecular weight and deacetylation degree.

**Figure 6:**
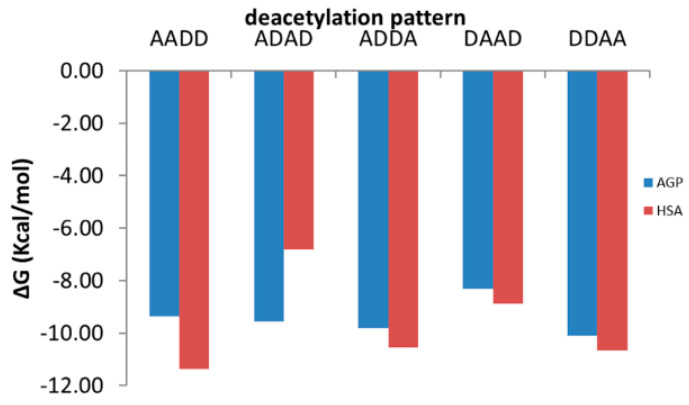
Binding energies of oligomers with similar molecular weight and deacetylation degree but distinct deacetylation patterns to AGP (blue columns) and HSA (red columns): the symbol A corresponds to a GlcNAc molecule and the symbol D to a GlcN molecule.

Figure 5 illustrate the dependence on the molecular weight (MW) and deacetylation degree (DD) of the interacting energies of investigated COs with AGP (Figure 5a) and HSA (Figure 5b) respectively and Figure 6 reveals the dependence of the interacting energies of investigated COs with AGP and HSA on deacetylation pattern.

Interacting energies of COs with both AGP and HSA increase with increasing molecular weight and increase with decreasing of deacetylation degree. For COs having similar molecular weights and deacetylation degrees, the binding energy depends on the deacetylation patterns (Figure 6). The interacting energies are usually higher for the COs interactions with HSA than with AGP. One-way ANOVA test implemented under ORIGINLab software illustrates that, at the 0.05 level, there are significantly different values for the binding energies to every of the two proteins and corresponding to various deacetylation degree and deacetylation patterns.

### 2.4. Characterization of Interactions 0f the Investigated Cos and the Two Plasma Proteins

**Figure 7:**
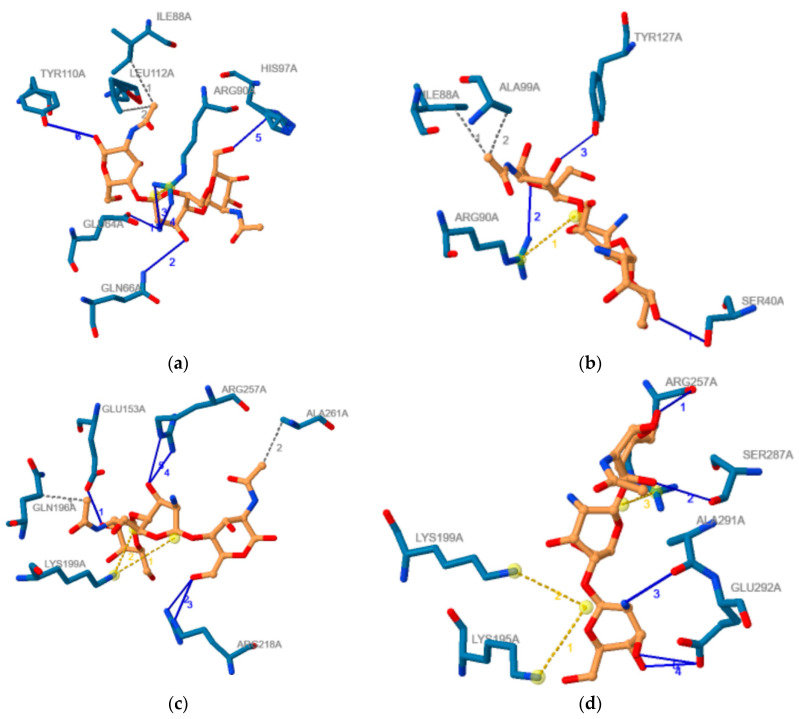
2D image of the noncovalent contacts between the ADDA and DADA chito-oligomers and AGP (**a**,**b**) and HSA (**c**,**d**), respectively. Blue lines illustrate hydrogen bonds, dashed grey lines illustrate hydrophobic contacts, and yellow dashed line illustrate salt bridges. If more than one noncovalent contact is made, than any type of noncovalent bod is numbered, numbers having the same color as the type they belong.

The outcomes obtained using PLIP software regarding the noncovalent contacts in the proteins–COs complexes obtained through molecular docking and corresponding to the most favorable binding modes are illustrated in Table 2. This table also contains the binding energies for these binding modes of investigated COs to AGP and HSA respectively. Furthermore, Figure 7 illustrate the 2D image of the noncovalent contacts between the ADDA and DADA oligomers and AGP (Figure 7a,b) and HSA (Figure 7c,d), respectively.

Data presented in Table 2 confirm the results emphasized by the molecular docking study. COs with higher molecular weight reveal a higher number of contacts in correlation with interacting energy that increases with molecular weight. For the same chito-oligosaccharide, the number of hydrophobic contacts and salt bridges is higher for the complex formed with HSA than with AGP and it corresponds to the higher interaction energies between COs and HSA. For COs with similar molecular weights and deacetylation degrees, but with distinct deacetylation pattern, the spectra of non-covalent bonds formed with every of the two plasma proteins are different, underlying the importance of this property of COs. Totally deacetylated COs does not make hydrogen bonds. Furthermore, the number of residues involved in the interactions of Cos with the two plasma proteins is usually higher than the number of AGP residues interacting to (2R)-2,3-dihydroxypropyl acetate and respectively than the number of residues of HSA interacting to DIF 1007 molecule, the ligands that are present in the crystallographic structures of the two molecules and those binding cavities correspond to binding poses of COs.

## 3. Discussion

The detailed investigation of plasma proteins and COs interactions is necessary to understand the pharmacodynamics and pharmacokinetics profiles of these molecules. The binding of COs to plasma proteins may act as a pool for a long duration of action of the molecules and may also affects the ADMET properties. The present study illustrates that there are favorable interactions between small chito-oligosaccharides and plasma proteins, AGP and HSA respectively, the interactions with HSA being stronger. The interactions of COs with AGP and/or HSA has a potential impact on their bioavailability, distribution, clearance, efficacy as antimicrobial agents and safety. COs bound to plasma proteins will not be available for the first pass metabolism, there is a lower volume of COs available to the target proteins and the clearance rate is decreased. Knowing the residues of AGP and HAS responsible for binding/stabilization of COs with various MW, DD, and DAP is important the fields of chemistry and clinical medicine as it allows designing COs with desired ADMET properties. Another consequence of COs binding to plasma proteins is their possible inhibitory effect against the interactions of these proteins with other compounds, being known that these proteins bind a wide diversity of endogenous and exogenous ligands [23].

These predictions obtained through structure-based molecular modeling may be further supported by experimental data. This is a promising integrated alternative strategy for ligand properties optimization, the use of molecular modeling combined with bioanalytical techniques being frequently used for the investigation of ligands binding to plasma proteins [24]. Many experimental techniques can be utilized to study the interactions of various xenobiotics with serum proteins. It is not the aim of this study to review such experimental approaches, but we enumerate few possibilities: (i) absorption, fluorescence, and/or nuclear magnetic resonance (NMR) spectroscopy; (ii) equilibrium dialysis; (iii) ultrafiltration; (iv) surface plasmon resonance; (v) capillary electrophoresis; (vi) X-ray crystallography; (vii) high-performance affinity chromatography [25]. To validate the results and add new information to the present study, these methods can be used to evaluate the average extent of binding of COs to plasma proteins, to determine the location and structure of the binding region of COs to plasma proteins, for the measurements of equilibrium constants, for assessing the effects of the various factors (temperature, pH, ionic strength, etc.) to protein-ligand binding and/or to determine the relative contributions of various factors to the formation and stabilization of the complex of protein–chitooligosaccharide.

## 4. Materials and Methods

In the present study, we have considered chito-oligosaccharides containing maximum six monomeric units and being characterized by various deacetylation degrees and patterns (see Table 1). Their simplified molecular-input line-entry system (SMILES) structures and the structural files in *mol* format were built using ACD/ChemSketch 2020 software [26]. This tool also computed the molecular weight of chito-oligosaccharides. Glucosamine (GlcN, D) have an amino group that is protonated at physiological pH [12] and consequently, in our computation, each amino group of a deacetylated unit is protonated.

Molecular docking is used to predict the noncovalent binding of investigated COs to plasma proteins. In order to implement this method, the three-dimensional structures of two proteins are necessary. Protein Data Bank (PDB) is the open access resource for protein structures [27] and we have used it to extract the structures of HSA and AGP. For AGP, the structural file with the PDB ID 3KQ0 has been considered because it is the single structural file of the protein without mutations and in complex with a ligand. For HSA, the crystallographic structure of the protein in complex with palmitic acid (PLM), diclofenac (DIF), and pentadecanoic acid (PA), having the PDB code entry 4Z69 has been taken into account [21]. This structural file has been chosen for HSA as the protein does not have mutations and there are multiple ligands bound in the three domains of the protein, allowing to obtain information regarding the preference in region of binding for investigated COs. Analysis of the structural files of AGP and HSA, respectively (as presented in Section 2.2) has been performed using Chimera 1.14 software (produced by Resource for Biocomputing, Visualization, and Informatics, University of California, San Francisco, USA) [28]. For molecular docking studies, we have used SwissDock web server (produced by Swiss Institute of Bioinformatics, Lausanne, Switerland) [29] computational tool. It uses the EADock algorithm [30] to compute the pairwise interaction energy between the ligand and the protein. The following steps have been considered when applying the molecular docking study: (i) we have extracted the structural files of the two proteins from the Protein Data Bank (in the case of HSA only the A chain of the structural file 4Z69 has been considered for molecular docking); (ii) the proteins and ligands were prepared for molecular docking (adding hydrogen atoms and considering charges) using Chimera 1.14 software; (iii) SwissDock web server has been used for implementing the molecular docking study and we have considered accurate, rigid, and blind docking; (iv) visualization and analysis of docking results have been performed using Chimera 1.14 software. 

The characterization of interactions of the investigated COs and the two plasma proteins has been made using Protein Ligand Interaction Profiler (PLIP) computational tool provided by Biotechnology Center TU Dresden (Germany) and that is freely accessible online [31]. This software has been used to detect the possible non-covalent contacts (hydrophobic contacts, hydrogen bonds, salt bridges, pi-stacking, pi-cation interactions, etc.) in the proteins–COs complexes obtained through molecular docking [32].

## 5. Conclusions

As an outcome of this study, we offered a structural depiction of where and how investigated chito-oligosaccharides bind to α-1-acid glycoprotein and human serum albumin such as to support the optimization of the ADME properties of COs related to plasma proteins binding, this being one of the factors determining the stability, distribution, metabolism, and toxicity of these compounds during therapeutic procedures. All investigated COs are able to bind to AGP and HSA, respectively. Interaction energies of COs with plasma proteins increase with increasing the molecular weight and decrease with increasing deacetylation degree. Furthermore, investigated COs reflect a stronger interaction with human serum albumin than with α-1-acid glycoprotein. For similar molecular weights and deacetylation degrees of COs, their interactions with plasma proteins are reliant on the deacetylation pattern. All these results illustrate that COs fate of distribution in the human organism is dependent on molecular weight, deacetylation degree, and deacetylation pattern. In addition, taking into account the dependence of binding energies on the deacetylation degree and deacetylation pattern, the preparation of chito-oligosaccharides with well-defined DD and DAP is required. These outcomes are useful as they inform the application of chito-oligosaccharides with varying molecular weights, degrees, and patterns of deacetylation in human health.

## Figures and Tables

**Table 1:** Chito-oligosaccharides considered in this study and their molecular weight (MW): GlcNAc or A is the acronym for N-acetyl-D-glucosamine, GlcN or D is the acronym for D-glucosamine.

Deacetilation Degree	Deacetylation Pattern	Acronym	MW (g/mol)
0%	GlcNAc	1A	221.21
	(GlcNAc)_2_	2A	424.40
	(GlcNAc)_3_	3A	627.59
	(GlcNAc)_4_	4A	870.79
	(GlcNAc)_5_	5A	1033.98
	(GlcNAc)_6_	6A	1237.17
33%	GlcNAc-GlcN-GlcNAc	ADA	585.56
50%	GlcN-GlcNAc	DA	382.36
	(GlcN-GlcNAc)_2_	DADA	746.71
	(GlcNAc-GlcN)_2_	ADAD	746.71
	GlcNAc-GlcNAc-GlcN-GlcN	AADD	746.71
	GlcN-GlcN-GlcNAc-GlcNAc	DDAA	746.71
	GlcN-GlcNAc-GlcNAc-GlcN	DAAD	746.71
	GlcNAc-GlcN-GlcN-GlcNAc	ADDA	746.71
	(GlcN-GlcNAc)_3_	DADADA	1475.41
	(GlcNAc-GlcN)_3_	ADADAD	1475.41
67%	GlcN-GlcN-GlcNAc	DDA	543.52
	GlcNAc-GlcN-GlcN-GlcN-GlcNAc-GlcN	ADDDAD	1069.02
	GlcN-GlcN-GlcN-GlcNAc-GlcN-GlcNAc	DDDADA	1069.02
100%	GlcN	D	179.17
	(GlcN)_2_	2D	340.33
	(GlcN)_3_	3D	501.48
	(GlcN)_4_	4D	662.64
	(GlcN)_5_	5D	823.79
	(GlcN)_6_	6D	984.95

**Table 2:** Illustration of the amino acids involved in the non-covalent contacts between COs and AGP and COs and HSA respectively, detected using PLIP software in the protein-ligand complexes corresponding to the most favorable binding modes resulting from molecular docking. The binding energies are also presented. In parenthesis is shown the number of contacts if it is higher than 1.

COs	Contacts with Alpha-1-Glycoprotein (AGP)				Contacts with Human Serum Albumin (HSA)			
	Binding Energy (kcal/mol)	Hydrophobic Interactions	Hydrogen Bonds	Salt Bridges	Binding Energy (kcal/mol)	Hydrophobic Interactions	Hydrogen Bonds	Salt Bridges
1A	−5.90	PHE49	SER89, ARG90, HIS97	ARG90	−6.70	LEU219	ARG257, SER287	none
1D	−5.47	none	TYR27, TYR127	ARG90	−4.89	none	ARG257 (2)	none
2A	−8.11	PHE49	SER30, GLU64, GLN66, ARG90, SER125 (2)	none	−8.69	LEU238, VAL241	LYS199, ARG218, ARG257, ILE290	none
2D	−7.66	none	ARG90	HIS97	−8.37	none	LYS199, ARG257 (3), SER287	LYS199, HIS242
DA	−7.94	PHE114	GLN66, ARG90 (2), SER125 (2), TYR127	none	−8.42	LEU219, ARG222, PHE223	LYS199, HIS242, ARG257, SER287	ARG257
3A	−8.75	TYR27, TYR37, ARG90, HIS97, PHE114 (2)	TYR37 (2), GLN66, ARG90, HIS97 (2)	ARG90, HIS97	−8.44	ARG218, LEU219, LEU238	SER192, LYS195, GLN196, LYS199, ARG218 (2), ARG257	LYS199
ADA	−8.15	ILE88, LEU112	GLU64, GLN66, ARG90 (2), HIS97, TYR110 (2)	ARG90	−10.28	GLN196, ALA261	GLU153, ARG218 (2), ARG257 (2)	LYS199 (2)
DDA	−8.74	ILE88, ALA99	SER40, ARG90, TYR127	ARG90	−9.89	none	ARG257, SER287, ALA291, GLU292 (2)	LYS195, LYS199, ARG257
3D	−5.36	none	GLU36, SER40	none	−5.74	none	TYR148, TYR150, GLN196 (2), GLU292	LYS195, ARG257
4A	−10.44	VAL41, ILE44, ILE88, GLN95, LEU112	GLU36, SER40, ARG90, GLY93, HIS97 (4)	ARG90, HIS97	−10.53	LEU260	GLU153, SER192, LYS195, GLN196, LYS199, ARG218, ARG257 (2), HIS288	LYS199, HIS288
AADD	−9.36	TYR27, ARG90, PHE114	SER40, ARG90, HIS97 (2), SER125 (2)	ARG90	−10.51	none	LYS195, ARG218, ARG222 (2), ARG257, SER287, ASP451	LYS195, LYS199, ARG218
ADAD	−9.56	TYR37, ARG90, PHE114	GLU36 (2), SER40 (2), THR47, GLN66, ARG90	none	−10.18	TYR452	GLU153, ARG160, GLU188, ARG218 (2), ARG222, ASP451	ARG160, LYS195, LYS281, HIS288
ADDA	−9.82	TYR27	SER40 (2), GLU64 (2), ARG90, HIS97	none	−11.62	GLU153, PHE157	ARG160, SER192, GLN196 (2), GLU292 (3)	LYS195, LYS199 (2), ARG257
DAAD	−8.31	ARG90, PHE114	SER30 (2), GLU36 (3), SER40, THR47, GLN66, ARG90, SER125 (2), TYR127	none	−7.32	GLN196	GLU153, LYS199, ARG218, HIS242, ARG257 (2), HIS288 (2)	LYS195, LYS199, ARG222
DADA	−6.11	ARG90, VAL92, PHE114	GLU64, ARG68 (2), ARG90, SER125, TYR127	ARG90	−6.89	PHE156	ARG160 (2), GLU184, GLU188, HIS288 (2), GLU292	ARG160 (2)
DDAA	−9.32	PHE49, PHE51, LEU112, TYR127	SER40 (3), ARG68 (2), HIS97, TYR127	ARG90	−10.34	TYR148, GLN196, ARG197	GLN196, LYS199, ARG257	ARG257
4D	−9.85	none	GLU36, SER40 (2), GLU64, ARG90, SER125 (2), TYR127	none	−11.25	none	GLU6, ARG10, GLU252 (3), ASP255	HIS3, LYS240
5A	−10.54	TYR37, ILE88, ALA99, LEU112	GLU36 (2), SER40, GLU64, ARG90, GLY93, HIS97	none	−13.53	LEU260	ARG160, LYS195, LYS199, ARG218 (2), ARG222, ARG257, ALA291, GLU292 (2), TYR452	LYS195, LYS199
5D	−6.77	none	TYR27, SER30, GLU36, SER40, ARG90, HIS97, SER125, TYR127	none	−6.81	none	TYR148, GLU153 (3), GLU188, SER192 (2), LYS199 (2), HIS242 (2), GLU292 (2)	ARG160, LYS195, LYS199
6A	−11.08	TYR37, ILE44, LEU79, ILE88, ARG90, PHE114	TYR37, SER40 (2), GLU64, ARG68 (2), ARG90, HIS97 (2), TYR127	ARG68, ARG90 (2)	−12.07	ALA191, GLN196	GLU153 (2), LYS195, LYS199, ARG218 (2), ARG222 (2), ARG257, HIS288 (3), GLU292 (3)	ARG160, LYS195, LYS199, LYS281, HIS288
ADADAD	−10.31	PHE32, TYR37, ARG90, VAL92, PHE114	SER30, GLU36 (2), TYR37, SER40 (4), GLU64, GLN66, ARG90, GLY93, HIS97 (2), SER125 (2)	none	−13.03	THR420	GLU505, THR506, HIS510, LYS524 (2), THR527	LYS524 (3)
DADADA	−9.62	TYR27, TYR37, ILE44	SER40, GLN66, ARG68 (2), TYR127	ARG90 (2)	−9.68	LEU260, ALA261	ARG160, SER192, LYS195, LYS199 (2), ARG218 (2), ARG222, ARG257 (2), GLU292 (3), VAL293	LYS195, LYS199
ADDDAD	−8.27	ALA99	GLU36, GLU43	ARG90	−8.34	LYS436, TYR452	GLU184, GLU188, HIS288 (2), GLU292 (3), LYS436 (2), TYR452 (2)	ARG160, LYS436
DDDADA	−8.21	PHE32, ALA99, PHE114	GLU36, SER40, GLN66, ARG90, SER125 (2), TYR127	none	−8.28	LEU260	GLU153, LYS199 (2), ARG218, SER287, HIS288 (3), ALA291, GLU292 (3)	LYS195, ARG257
6D	−9.41	none	GLU36, SER40 (2), GLU64, ARG90, ASN117, ASP118, ASN121	ARG90, HIS97	−13.74	none	GLU153 (2), ARG160 (2), GLU188, LYS195 (2), LYS199, ARG218, GLU292	ARG160, LYS195, LYS199 (2)

## Data Availability

Not applicable.
